# Psychiatric rehabilitation patterns in Italy: Results from the Italian Society of Psychosocial Rehabilitation (SIRP) survey

**DOI:** 10.3389/fpsyt.2023.1130811

**Published:** 2023-02-22

**Authors:** Caterina Viganò, Cassandra Ariu, Deborah Barbieri, Alessia Goffredi, Luca Ferrara, Federico Rea, Stefano Barlati, Antonio Vita, SIRP Survey Group

**Affiliations:** ^1^Department of Biomedical and Clinical Sciences, University of Milan, Milan, Italy; ^2^Psychiatry 2 Unit, ASST Fatebenefratelli Sacco Milan, Milan, Italy; ^3^Department of Mental Health and Addiction Services, ASST Spedali Civili of Brescia, Brescia, Italy; ^4^ASST Ovest Milanese, Ospedale Vecchio di Legnano, Legnano, Italy; ^5^National Centre for Healthcare Research & Pharmacoepidemiology, University of Milano-Bicocca, Milan, Italy; ^6^Department of Clinical and Experimental Sciences, University of Brescia, Brescia, Italy

**Keywords:** case management (CM), community-based intervention, evidence-based, mental health services, psychosocial rehabilitation, recovery, severe mental illness (SMI)

## Abstract

**Introduction:**

Psychiatric rehabilitation can be considered a bidirectional technique, designed to allow patients to achieve their personal target, focusing on the individuals’ strengths and challenges related to these targets and also on the community organizations in which they will live them out. Unfortunately, psychiatric rehabilitation is too often not considered a first line treatment. Moreover, rehabilitation has been confused with a generic and rough practice, consisting of extemporary actions and aimless entertainments designed to fill “the time passing”.

**Methods:**

The aim of this study was to increase the knowledge and awareness about the state of the art of different systems of management and funding of psychosocial rehabilitation in the Italian “real-world” rehabilitative settings, using a specifically developed questionnaire.

**Results:**

The data obtained are positive for some aspects of the rehabilitation interventions, in particular for the use of validated tools for the evaluation and revision of projects and for the trend to work on a team, even though the scarcity of evidence-based rehabilitation interventions applied in Italian psychiatric services is less encouraging.

**Conclusion:**

This survey presents, at least partially, the “real-world” of rehabilitation in Italy so that we can lay the foundations for the definition of an updated, validated and shared network of what is implemented in the context of psychiatric rehabilitation.

## Introduction

In the second half of the 20th century, mental health care in all European and other high-income countries was revolutionized in its structure and conceptualization. Indeed, deinstitutionalization could take place after the introduction of antipsychotic agents in the 1950s and the consequent improvement of the treatment of schizophrenia and other psychotic disorders (1, 2). Of course this change allowed to reduce the number of hospitalizations and, for the first time, gave priority to outpatient care and community-based services that sprout in different community mental health programs across and within these countries (3, 4). However, there has been a growing but controversial literature about the outcomes achieved through antipsychotic medication alone, especially in terms of functional recovery (5–8). It is also necessary to take into consideration medication non-adherence, which unfortunately involves more than half of patients (9–11) and therefore compromises efficacy rates. In order to fill this gap, several non-pharmacological interventions have been developed for severe mental illnesses (SMI). In particular, psychiatric rehabilitation can be considered a bidirectional technique, designed to allow patients to achieve their personal target, focusing on the individuals’ strengths and challenges related to these targets and also on the community organizations in which they will live them out (12, 13). Psychiatric rehabilitation practices should be multidimensional and useful in the majority of SMI, with the primary goal to personalize and integrate treatment, trying to address multiple health and diseases related variables (14, 15). As reported by the World Psychiatric Association (WPA), the aim of psychosocial rehabilitation is to help individuals suffering from severe and persistent mental disorders to develop the intellectual, emotional, and social skills and conditions necessary to live, learn, and work in the community to which they belong with the minimum amount of professional support. Psychosocial rehabilitation interventions are based on the assumption that, regardless of their clinical severity, a patient’s functioning areas can always be improved by therapeutic and rehabilitation work (16, 17). Unfortunately, psychiatric rehabilitation is too often not considered a first line treatment and it is used only once other types of intervention have failed. Moreover, rehabilitation has been confused with a generic and rough practice, consisting of extemporary actions and aimless entertainments designed to fill “the time passing” (12, 17). In recent years, however, psychiatric rehabilitation practice has been gradually refining and consolidating its paradigms, offering structured and progressive interventions with selective and targeted objectives, defined by specific procedures of assessment and evaluation (18, 19). Researchers identified a large and growing number of effective psychiatric rehabilitation practices (13, 19–21). Evidence-based models have been developed for many recovery goals, including employment, independent living and community living skills (22–24). They include for example: Assertive Community Treatment (ACT), Cognitive-Behavior Therapy (CBT) for psychosis, cognitive remediation (CR), family psychoeducation, illness self-management training, Social Skills Training (SST), and supported employment (18, 25–28). The term “evidence-based practice” refers to those interventions shown to be effective at improving the course or outcome of a specific illness, based on rigorously conducted research studies (29). For serious mental illnesses, there is a consensus that evidence-based practices require standardized interventions that should also be supported by multiple randomized controlled trials (RCTs) (at least two of which conducted by different research teams), having as target outcomes relevant to symptoms or impaired functioning (30). Furthermore, other psychosocial interventions are diffused and widely applied in Europe and also in Italy, although not yet evidence-based. Moreover, some data have shown that the additional use of art therapies and, in general of the so-called expressive therapies, reduced negative symptoms among schizophrenia patients and so it may allow the implementation of self-knowledge, awareness, relational competence and affective development in this population (31–40). It is also essential to emphasize the two main pillars of psychosocial rehabilitation: the first is that all the actors involved in the rehabilitative process must act in a consistent manner in order to optimize the treatment, and the second is that, even in different contexts, continuity must be assured by the same therapeutic team (41). On this issue, the continuity of care in the usual subject’s life context and the multidisciplinary care represent the approaches to psychiatric care of severe mental disorders prototypically implemented in Italy after the Psychiatric Reform of 1978 (42). Despite the growing scientific literature on the effectiveness and efficacy of this kind of rehabilitative interventions, they are not widely available in real-world practice. In fact, it is widely accepted that one of the most relevant problems in the public mental health system is the gap between scientifically effective practices and those actually used in mental health services (MHS) (43). Although these interventions produced promising results, there is still no consensus on their usefulness and applicability in clinical practice. High-income countries in Europe continued to invest in costly traditional services that were neither evidence-based, nor person-centered by emphasizing inpatient services, sheltered group homes, day treatment centers and sheltered workshops (44). Only a minority of patients received appropriate outpatient treatments and evidence-based psychiatric rehabilitation interventions (45–47). One fundamental difficulty in developing community-based care has been that patients and professionals often disagree over the nature of mental disorders and the goals of treatments. Physicians and professionals often try to achieve clinical stability (symptoms control) as a primary goal instead of focusing on the quality of life of people with mental illness, who barely live satisfying and meaningful lives (48). This discrepancy could be filled by an evidence-based, person-centered, recovery-oriented psychiatric rehabilitation. The difficulty to implement effective psychiatric rehabilitation services in high-income countries is not primarily a lack of resources, but rather a lack of political will and inefficient use and dysfunctional allocation of resources (19, 44). The situation is not so different in the USA and, although the USA has led the way in developing, researching and disseminating evidence-based psychiatric rehabilitation services around the world, the quality of mental health rehabilitation services lags behind MHS in many other industrialized nations (22). Despite the wide endorsement of recovery as a guiding principle of psychiatric rehabilitation (49), state mental health agencies in the USA continue to support outdated psychosocial services, such as brokered case management services, day treatment and institution-based skills training. Moreover, mental health spending includes consistent allocations of funding for psychotropic drugs, completely unbalanced with respect to funding allocated for psychosocial recovery-oriented interventions (22). Furthermore, other barriers for the dissemination of psychiatric rehabilitation interventions could also be the lack of sufficient evidences regarding their specific indications, mechanisms of action, adverse effects, but also practical issues concerning the interpretability of respective clinical studies, such as the choice of outcome variables and control of confounding factors (1). The existing literature on psychiatric rehabilitation delivery is still scarce all over the world and, to our knowledge, no specific assessment has been performed on psychosocial interventions delivery in the Italian mental health real-world settings. In particular, the operational translation of the psychiatric rehabilitation evidence and its theoretical concepts have never been the object of a careful analysis in order to verify which rehabilitation activities and techniques were actually offered in the Italian mental health delivery system. There is only a previous survey limited to a northern Italian area (Lombardia region) carried out by the Italian Society for Psychosocial Rehabilitation (SIRP), with the aim of evaluating the kind of activities actually implemented in Italy (50). Current psychosocial rehabilitation practices are highly variable in terms of methodology and contents, with relevant differences from one region to another, and also within the same region, according to the specific orientation and tradition characterizing each Department of Mental Health.

The aim of this study was to increase the knowledge and awareness about the state of the art of different systems of management and funding of psychosocial rehabilitation in the Italian “real-world” rehabilitative settings, using a specifically developed questionnaire.

## Materials and methods

This research project was carried out by the Italian Society of Psychosocial Rehabilitation (SIRP), a scientific society, which aims to promote personal, social, and professional rehabilitation of people with SMI and their families. The Survey was planned in two phases; the first stage (performed around 2014) involved creating a “SIRP Survey Team”, a multi-professional national group composed by members of the Board of Directors of SIRP: professional educators, psychiatric rehabilitation technicians, psychologists and psychiatrists. Firstly they reviewed and adapted the questionnaire and other materials of a previous pilot project carried out in Lombardia to a national reality (50).

The second step occurred between March and May 2015, consisting of the distribution and the collection of the questionnaire. In 2015, SIRP sections were present in 15 Italian regions that were all involved in the project. This operational phase has been characterized in building and sending the project cover letters, in paper and/or digital form, to the regional SIRP offices and then to the Departments of Mental Health of each region. Then the questionnaire was directly presented to the Directors and/or the main contacts of the rehabilitation facilities. Finally, a database was created for the collection and the analysis of data, which needed a further revision to catch incomplete or contradictory data before the definitive investigation, by directly contacting the referring facilities involved, in order to better categorize the rehabilitation activities.

### Instruments

The former discussed questionnaire was developed with the aim to investigate rehabilitation activities in Italian MHS; it was composed mostly by closed questions and multiple-choice answers. It included a first descriptive part specifically oriented at depicting the service participating at the survey and a second part, in which single “activity forms” were used to graphically explain each rehabilitative activity. The initial section of the questionnaire was dedicated to the relevant data of the MHS: region, province, Operative Unit, Department of Mental Health, Hospital/Local Health Service catchment area, type of facility, application of the Individual Treatment Plan (PTI) and of the Rehabilitation Therapeutic Project (PTR) (51), as well as the use of outcome assessment tools. The second section consisted of the descriptive cards and needed to be filled in by the operator who had conducted the activity, who had to describe the activity and how it was done, together with the contents and the techniques used, besides indicating the name of the activity. Then the type of activity as provided in the National Mental Health Information System (SISM) (52) should have been indicated. It was also required to indicate the number and professional qualifications of the operators involved (including volunteers, organizations and associations) and the number of average users per session. The final part of the card showed the place where the activity had taken place (in the facility or at home, in a workshop, gym or others) and its weekly frequency and duration (periodical or continuous). Finally, to support the descriptive open part, the SIRP group supplied to the questionnaire a list of specific categories of activities. This list was created because the exclusive use of the SISM categorization (Table 1) appeared to be too specific to describe better, for example, the concept of “rehabilitation intervention with respect to basic interpersonal skills”, that includes multiple activities aimed at developing skills of independent living in everyday life. Therefore, the SIRP workgroup prepared a list of 20 “categories” of rehabilitation activities that seemed to sufficiently represent most of the rehabilitation interventions applied in the various Italian services, also starting from what already existed in the Italian and international literature (Table 2; 53, 54). This list of categories, compiled by the Survey SIRP workgroup, was attached to the survey form, in order to allow the operator involved to classify the activity in a more descriptive way.

**TABLE 1:** Frequency of the SISM type of activity for each facilities.

	SISM categories	Frequency	Percentage		SISM categories	Frequency	Percentage
Semi residential facilities	Group re-socialization	156	18.9%	Supportive housing	Group re-socialization	19	19.4%
	Basic interpersonal group skills	125	15.1%		Daily life	14	14.3%
	Motorial interventions	114	13.8%		Group expressive skills	13	13.3%
	Group expressive skills	111	13.4%		Practical-craft intervention	12	12.2%
	Practical-craft intervention	88	10.6%		Basic interpersonal individual skills	11	11.2%
	Work training and coaching	52	6.3%		Basic interpersonal group skills	6	6.1%
	Basic interpersonal individual skills	35	4.2%		Vacation stay	4	4.1%
	Other activities for context	32	3.8%		Administrative and social problems	3	3.1%
	Psychoeducation	24	3.0%		Individual re-socialization	3	3.1%
	Individual expressive skills	20	2.4%		Motorial interventions	2	2.1%
	Daily life	14	1.7%		Psychoeducation	2	2.0%
	Vacation stay	14	1.7%		Other activities for family members	2	2.0%
	Other activities for family members	11	1.3%		Counseling of family members	2	2.0%
	Administrative and social problems	1	1.3%		Work training and coaching	1	1.1%
	Counseling of family members	9	1.1%		Other activities for context	1	1.0%
	against the stigma	7	0.8%		individual expressive skills	1	1.0%
	Administrative and social problems	6	0.7%		Against the stigma	1	1.0%
	Network interventions	6	0.7%		Network interventions	1	1.0%
	Individual re-socialization	4	0.5%				
Mental Health Center	Group re-socialization	17	21.2%	Residential Facilities	Group re-socialization	195	17.5%
	Basic interpersonal group skills	13	16.2%		Basic interpersonal group skills	160	14.4%
	Motorial interventions	11	13.7%		Group expressive skills	147	13.2%
	Practical-craft intervention	8	10.0%		Motorial interventions	128	11.5%
	Group expressive skills	6	7.4%		Practical-craft intervention	105	9.4%
	Basic interpersonal individual skills	5	6.3%		Basic interpersonal individual skills	78	6.9%
	Psychoeducation	3	3.8%		Daily life	61	5.5%
	Individual expressive skills	2	2.5%		Work training and coaching	45	4%
	Other activities for context	1	1.3%		Other activities for context	41	3.7%
	Daily life	1	1.3%		Psychoeducation	41	3.7%
	Against the stigma	1	1.3%		other activities for family members	20	1.8%
Psychiatric diagnosis and care service facility	Group re-socialization	7	53.8%		Counseling of family members	20	1.8%
	Work training and coaching	2	2.5%		Vacation stay	18	1.6%
	Individual re-socialization	1	1.3%		Individual expressive skills	17	1.5%
	Counseling of family members	2	2.5%		Individual re-socialization	17	1.5%
	Network interventions	2	2.4%		Administrative and social problems	9	0.8%
	Vacation stay	4	5.0%		Network interventions	9	0.8%
	Motorial interventions	2	15.4%		Against the stigma	5	0.4%
	Group expressive skills	1	7.7%				
	Network interventions	1	7.7%				
	Psychoeducation	1	7.7%				

**TABLE 2:** SIRP activity categories.

SIRP activity categories
Physical activity: not structured sports activities
Craft workshops: decoration, carpentry, sewing, cardboard techniques, leather, clay, graphic and painting activities with the objective of producing handmade products
Leisure, free time: organized outdoor activities: excursions, museums, exhibitions, movies, vacations
Daily activities organization/Support: self-care and environment care, money management
Sports: soccer, volleyball, sailing, various tournaments
Cooking: cooking group, cooking basics, organizing food shopping
Gardening and horticultural therapy activities
Music, singing, theater Group with socializing value
Re-socialization: all the occupational activities that are not part of specific groups, playing activities
Writing, stories, media: newspaper editorial, radio program management, media
Work: support activities and preparation for work placement (e.g., IPS)
Expressive techniques: art therapy, music therapy, dance therapy, dramatherapy, others expressive
Group aimed at the verbal self: supporting psychotherapies, discussion and autobiography
Cognitive remediation
Social skills training
Psychoeducation for patients
Other structured psychoeducational program: informative/problem solving modules, wellness groups
Animal assisted therapy
Activities for family members (from counseling to psychoeducation)
Others: e.g., self-help, activities against stigma, etc.

IPS, individual placement and support; IPT, integrated psychological therapy; SIRP, Italian Society for Psychosocial Rehabilitation.

### Sample

The project cover letters were sent to the 15 regional SIRP offices and then to the Departments of Mental Health of each region. The final sample was composed by 4.611 institutions in 176 Departments of Mental Health: 1.221 Mental Health Center, 890 Day-time Center and Day Hospital, 2.181 Residential Facilities, 319 Psychiatric Diagnosis and Care Service facilities. The first part of the questionnaire was filled in by the structure heads, whereas the second part by the responsible operator for each activity. The services involved in the survey were then asked to fill in a single form for each structured rehabilitation activity carried out continuously for at least 12 months between 2012 and 2015. Finally, each facility provided a descriptive part of the structure and several activity cards. The method used was non-probability sampling.

### Statistical analysis

Summary statistics of the types of facilities and characteristics of the activities performed were expressed as mean (minimum-maximum), or counts and percentage, as appropriate.

The Statistical Analysis System Software (version 9.4; SAS Institute, Cary, NC, USA) was used for the analyses, with the support of the Biostatistics, Epidemiology and Public Health Unit of the Milan Bicocca University.

## Results

Our survey has represented 10 regions, which were 66.6% of those related to the SIRP in that period. The total number of usable activity forms among the received ones was 2.255 out of 3.553, since they were completed with the description of the rehabilitative activities. Analyzing the MHS response rate we can observe that 41 Departments of Mental Health responded to the survey, equal to 23.3%, with 418 Italian facilities that wrote up the questionnaire, equal to 9.1% of the units part of the Italian Mental Health Departments linked to the SIRP in that period (according to data from SISM Mental Health Report 2016), published in December 2016 by the Ministry of Health. Furthermore, we looked at the percentage of activity forms received according to different types of services: residential facilities collected 49.7% of activity forms, with 1.120 cards (73.8% with 24-hours assistance, 14% with 12-h assistance, 12.1% other residential services and 0.1% with 6-h assistance), followed by semi-residential facilities, 36.8% with 829 activity forms (93.5% day-time centers, 4.3% day hospital, 2.2% other semi-residential facilities). Mental Health Centers collected 3.5% of activity forms, with 80 cards, and Psychiatric Diagnosis Care Service collected 0.6% of activity forms, with 13 cards. Finally, Supportive Housing collected 4.3% of activity forms, with 98 cards, and “others facilities” collected 5.1% of activity cards, with 115 cards.

### Criteria and procedures in the creation of the rehabilitation project

The rehabilitation project in Italy is evaluated through the use of tools such as the PTI (Individual Therapeutic Plan) drawn up by the Mental Health Centers sending team, and the PTR (Rehabilitation Therapeutic Project), which is a development of the patient’s PTI in rehabilitation facilities, drawn up by the team receiving the patient him/herself (51). In our survey both the PTI and the PTR were used in the definition of the rehabilitation program in more than half of the cases (58.3%), whereas 24.1% of the operators used only PTR and 9.1% used only PTI (8.5% were missing data). Eventually, 88.4% made a revision of the rehabilitation projects. This review of the project was actuated in 48.6% of the cases with times planned to be customized to the needs of the individual patient, whereas in 39.2% of the cases the revision was operated on standardized times. Only in 0.9% of the facilities there was no revision of the rehabilitation program. The remaining 11.3% of cases were not evaluated. Examining the details of these data, we found out that in semi residential facilities the revision of the project was often customized (63.3%), whereas in residential facilities the project was reviewed in 47.2% in standardized time and in 42.2% in customized modality (10.6% were missing data). Concerning the patient’s evaluation, we examined how many and which facilities completed it and which areas they evaluated between psychopathological, functioning, and others (e.g., quality of life, cognition, social support, etc.). By the analysis, it emerged that 68.0% of the structures used evaluation tools, whereas 21.9% did not take into account the assessment of the patients (10.1% were missing data). The data were similar for all the facilities, with the exception of the Psychiatric Diagnosis Care Service, where no data emerged regarding the use of evaluation scales (33.3% did not evaluate, 66.7% did not answer the question).

Concerning the use of assessment scales, in the facilities performing the evaluation in the Italian regions, 35.4% investigated the psychopathological dimension, 38.9% investigated social functioning and 57.9% investigated other areas of patient functioning. In detail, psychopathological areas were more evaluated in residential facilities (42.2%), whereas social functioning was evaluated with a similar percentage both in residential facilities (46.5%) and in Mental Health Centers – MHC (41.3%). 100% of supporting housing didn’t consider this area. There were found no differences concerning “other areas” that were evaluated, with similar rates in all the facilities.

### Description of activities

Through the analysis of the questionnaires received, we looked closely at the average number of users involved per session, the place of the activity (on site, at the patient’s home or elsewhere) and the duration of the activity (that could have been continuous, forward or cyclical). Hereinafter there is an analysis of the overall data divided by type of structure. Complete data are available in the Supplementary Table 1.

Concerning the 2.255 activities examined, the national data showed that 3.5% involved an average of 1 user per session, 21.6% an average of 2 to 5, 41.1% an average of 6 to 10, 25.1% an average of 11 to 20 and 6.7% more than 20. Finally, 2% were missing data (see Supplementary Table 2). In particular, MHC involved 6 to 10 users in 38.8% of the cases, whereas in 26.3% of cases 11 to 20 users per meeting. Residential facilities behaved similarly, including 6 to 10 users in 42.1% of cases and 11 to 20 users in 27.5% of cases.

Regarding semi-residential facilities, they include in 43.7% of cases from 6 to 10 users and in 25.1% of cases from 11 to 20 users. Similar data were also observed in the Psychiatric Diagnosis Care Service, which in 61.5% of cases involved 6 to 10 users per session, while in 23.1% of cases it involved an average superior to 20. As regards Supportive Housing, the facilities differed from the other structures: 82.7% of the activities involved 2 to 5 users per meeting.

With respect to the place where the activities took place, 60.4% of activities were carried out in the facilities, 32.2% in other places and 0.5% at the patient’s home. The remaining activities were conducted in multiple locations (both on-site, at home or elsewhere). In particular, 58.8% of the activities of the MHC were carried out outside the facilities, 36.3% indoors and 1.3% at the patient’s home. 63.7% of the activities in the residential structures occurred inside the facilities and 31.6% in other places, just as 59.6% happened in the semi-residential unit (30.4% in other places) and 69.2% of the activities of the psychiatric acute ward (23.1% elsewhere). Always concerning the Supportive Housing, 43.9% of the activities were carried out indoors and 36.7% outside the facilities. The data relating to the patient’s home and other places used can be assimilated for each structure to the overall data (see Supplementary Table 1.

Later on, regarding the duration of the activities, we discovered that 61.5% of them were carried out within the structure continuously, 19.2% in a cyclical way and 16.8% with a term. The remaining were missing data. In detail, it can be observed that within the MHC, 61.3% of activities took place continuously and 25.0% on a fixed-term basis, as well as in residential structures (66.8% in continuous, 17.0% forward and 15.4% cyclically). However, concerning semi-residential activities, 54.6% happened continuously, whereas 23.4% occurred cyclically and 17.4% on a temporary basis. Within the Psychiatric Diagnosis Care Service, 53.8% of the activities were carried out continuously and the remainder were conducted for 23.1% on a term or cyclical basis. Furthermore, always with regard to Supportive Housing, 75.5% was carried out continuously and 12.2% at term. A Supplementary material is available to consult the remaining data (Supplementary Table 1).

### Activities described according to the SISM classification

Using the SISM classification (52), the activities were split up in 19 categories: activities addressed to patients (basic interpersonal individual skills, basic interpersonal group skills, individual re-socialization, group re-socialization, vacation stay, individual expressive skills, group expressive skills, motorial interventions, practical-craft intervention, work training and coaching, daily life, administrative and social problems), activities addressed to family members (counseling of family members, psychoeducation and other activities for family members) and addressed to the context (network interventions, against the stigma and other activities for context).

From the analysis emerged that 18.3% of the described activities were group re-socialization activities and 14.1% aimed at stimulating basic interpersonal skills (group), followed by group expressive skills 12.9 and 12.0% motorial interventions. Practical- craft interventions were 9.9%, whereas basic interpersonal skills (individual) were 6.0%.

For the leftover categories see the Supplementary Table 3.

For each facility involved, it was examined the frequency of activities performed (Table 1). Unspecified facilities (other and mixed) were not included in this analysis.

The analysis also showed that residential facilities employed a group modality in 62.3% of the cases, an individual modality in 18.5% and a mixed modality in 19.2%. Similarly, in semi-residential facilities, where group activities represented 68.4% of the total, individual activities were 14.5% and mixed activities were 17.1%. In the Mental Health Center, group activities were 62.8%, meanwhile individual activities were 19.2% and mixed activities represented 17.9%. In the same way, in Psychiatric Diagnosis and Care Service units groups activities represented 69.2% of the total, whereas individual ones were 15.4% and mixed modality activities were 15.4%. Finally, the Supportive Housing employed in 49.5% of cases a group modality, in 23.7% of cases an individual one and in 26.8% of cases a mixed modality.

### Frequency of activities according to the SIRP descriptive categories

Proceeding on the investigation, we conducted our analysis using the Survey SIRP workgroup’s list, instead of SISM categories. Table 2 shows how activities were split up.

**FIGURE 1:**
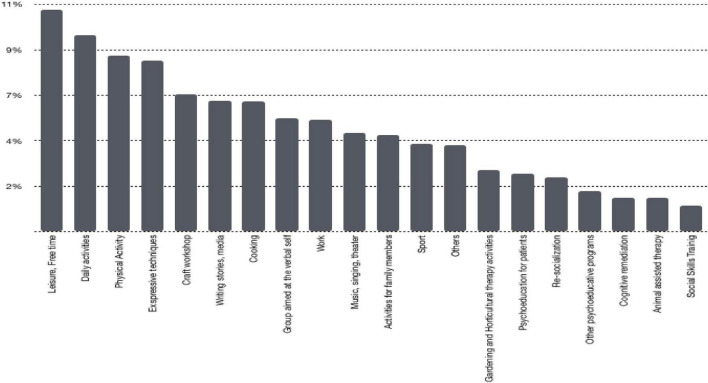
National distribution of the frequency of categories related to the rehabilitation activities, according to Italian Society for Psychosocial Rehabilitation (SIRP) categorization.

According to this list, the type of activity most frequently performed in the centers involved in the Survey was the one addressed to the leisure activities management (with 10.7%), followed by activities in support of daily life with 9.5% (self-care, environment care, money management, etc.), physical training activities in the facility (physical training group with 8.5%) and expressive techniques with 8.3%. Furthermore, it shows that craft workshops represented 6.6% of the activities, meanwhile writing stories media represented 6.4% and cooking represented 6.3% of the total. For the remaining activities see Figure 1. Specific data are available in the Supplementary Table 4.

Concerning each activities, we examined the number of the operators involved, their frequency and their carrying out modality (group, individual or both). Table 3 describes all the variables investigated for every activity. For additional data see the Supplementary Table 5.

**TABLE 3:** Description of activities according to SIRP classification.

Activity	Facility	%	Frequency	%	Administration way	%
Physical activity	Semi residential facilities	35.2%	Monthly	6.3%	Individual	4.2%
	Mental health center	3.6%	Bi-weekly	28.8%	Group	32.3%
	Psychiatric diagnosis and care service facility	1.1%	Weekly	56.0%	Both	63.5%
	Supportive housing	2.6%	Other	8.9%	MHW involved (medium)	2.3
	Residential facilities	53.4%				
	Others	4.1%				
Craft workshops	Semi residential facilities	45.4%	Monthly	1.4%	Individual	8.7%
	Mental health center	5.3%	Bi-weekly	26.4%	Group	65.3%
	Psychiatric diagnosis and care service facility	0.7%	Weekly	52.7%	Both	26.0%
	Supportive housing	1.3%	Other	19.6%	MHW involved (medium)	2.7
	Residential facilities	42.0%				
	Others	5.3%				
Free time	Semi residential facilities	34.3%	Monthly	19.5%	Individual	5.4%
	Mental health center	6.6%	Bi-weekly	6.4%	Group	90.5%
	Psychiatric diagnosis and care service facility	0.4%	Weekly	31.8%	Both	4.1%
	Supportive housing	7.9%	Other	42.4%	MHW involved (medium)	3.1
	Residential facilities	45.4%				
	Others	5.4%				
Daily activities organization	Semi residential facilities	17.2%	Monthly	5.2%	Individual	39.7%
	Mental health center	1.9%	Bi-weekly	14.6%	Group	35.5%
	Psychiatric diagnosis and care service facility	0.5%	Weekly	34.3%	Both	24.8%
	Supportive housing	10.2%	Other	46.0%	MHW involved (medium)	3.6
	Residential facilities	67.4%				
	Others	2.8%				
Sports	Semi residential facilities	48.9%	Monthly	5.5%	Individual	5.2%
	Mental health center	7.3%	Bi-weekly	17.6%	Group	51.0%
	Residential facilities	37.5%	Weekly	65.9%	Both	43.8%
	Others	6.3%	Other	11.0%	MHW involved (medium)	2.8
Cooking	Semi residential facilities	28.8%	Monthly	1.4%	Individual	15.5%
	Mental health center	2.2%	Bi-weekly	13.7%	Group	54.2%
	Supportive housing	10.6%	Weekly	51.1%	Both	29.6%
	Residential facilities	55.6%	Other	33.8%	MHW involved (medium)	2.7
	Others	2.8%				
Gardening	Semi residential facilities	34.3%	Monthly	3.1%	Individual	11.9%
	Mental health center	4.5%	Bi-weekly	26.6%	Group	44.8%
	Supportive housing	3.0%	Weekly	35.9%	Both	43.3%
	residential facilities	55.2%	Other	34.4%	MHW involved (medium)	1.9
	Others	3.0%				
Music, singing, theater	Semi residential facilities	42.6%	Monthly	4.7%	Individual	11.1%
	Psychiatric diagnosis and care service facility	1.8%	Bi-weekly	14.2%	Group	88.0%
	Supportive housing	0.9%	Weekly	75.5%	Both	0.9%
	Residential facilities	50.9%	Other	5.7%	MHW involved (medium)	2.5
	Others	3.8%				
Resocialization	Semi residential facilities	40.7%	Monthly	3.4%	Individual	5.1%
	Residential facilities	57.6%	Bi-weekly	17.2%	Group	89.8%
	Others	1.7%	Weekly	43.1%	Both	5.1%
			other	36.2%	MHW involved (medium)	3.3
Writing, stories, media	Semi residential facilities	44.7%	Monthly	3.6%	individual	8.4%
	Mental health center	1.4%	Bi-weekly	15.2%	Group	88.8%
	Psychiatric diagnosis and care service facility	0.7%	Weekly	66.7%	Both	2.8%
	Supportive housing	0.7%	Other	14.5%	MHW involved (medium)	2.7
	Residential facilities	48.9%				
	Others	3.6%				
Work	Semi residential facilities	50.8%	Monthly	4.2%	Individual	50.0%
	Mental health center	2.5%	Bi-weekly	19.5%	Group	28.7%
	Supportive housing	1.6%	Weekly	27.1%	Both	21.3%
	Residential facilities	41.0%	Other	49.2%	MHW involved (medium)	2.1
	Others	4.1%				
Expressive techniques	Semi residential facilities	39.3%	Monthly	3.3%	Individual	16.1%
	Mental health center	3.2%	Bi-weekly	11.5%	Group	82.3%
	Psychiatric diagnosis and care service facility	0.5%	Weekly	75.4%	Both	1.1%
	Supportive housing	1.6%	Other	9.8%	MHW involved (medium)	3.2
	Residential facilities	50.0%				
	Others	5.4%				
Group aimed at the verbal self	Semi residential facilities	41.1%	Monthly	4.1%	Individual	8.9%
	Mental health center	2.4%	Bi-weekly	9.0%	Group	86.3%
	Psychiatric diagnosis and care service facility	1.6%	Weekly	74.6%	Both	3.2%
	Supportive housing	11.4%	Other	12.3%	MHW involved (medium)	2.9
	Residential facilities	41.1%				
	Others	2.4%				
Cognitive remediation	Semi residential facilities	32.5%	Monthly	8.1%	Individual	32.4%
	Residential facilities	54.0%	Bi-weekly	32.4%	Group	67.6%
	Others	13.5%	Weekly	48.6%	Both	0.0%
			other	10.8%	MHW involved (medium)	2.4
Social skills training	Semi residential facilities	32.1%	Monthly	0.0%	Individual	10.7%
	Mental health center	3.7%	Bi-weekly	10.7%	Group	89.3%
	Supportive housing	7.1%	Weekly	82.1%	Both	0.0%
	Residential facilities	50.0%	Other	7.1%	MHW involved (medium)	2.5
	Others	7.1%				
Psychoeducation for patients	Semi residential facilities	25.4%	Monthly	17.5%	Individual	15.9%
	Mental health center	6.3%	Bi-weekly	3.2%	Group	54.0%
	Supportive housing	3.2%	Weekly	60.3%	Both	30.2%
	Residential facilities	60.3%	Other	19.0%	MHW involved (medium)	3.1
	Others	4.8%				
Other structured psychoeducational program	Semi residential facilities	34.1%	Monthly	14.0%	Individual	13.6%
	Mental health center	2.3%	Bi-weekly	18.6%	Group	84.1%
	Residential facilities	54.5%	Weekly	58.1%	Both	2.3%
	Others	9.1%	Other	9.3%	MHW involved (medium)	2.2
Animal assisted therapy	Semi residential facilities	37.8%	Monthly	2.7%	Individual	21.6%
	Mental health center	5.4%	Bi-weekly	13.5%	Group	75.7%
	Residential facilities	43.3%	Weekly	64.9%	Both	2.7%
	Residential facilities	5.4%	Other	18.9%	MHW involved (medium)	2.5
	Others	8.1%				
Activities for family members	Semi residential facilities	32.3%	Monthly	45.8%	Individual	45.7%
	Mental health center	4.8%	Bi-weekly	9.4%	Group	47.6%
	Psychiatric diagnosis and care service facility	1.9%	Weekly	12.5%	Both	6.7%
	Supportive housing	5.7%	Other	32.3%	MHW involved (medium)	3
	Residential facilities	48.6%				
	Others	6.7%				
Others	Semi residential facilities	43.6%	Monthly	9.0%	Individual	21.3%
	Mental health center	5.3%	Bi-weekly	6.7%	Group	68.1%
	Residential facilities	34.1%	Weekly	28.1%	Bth	10.6%
	Others	17.0%	Other	56.2%	MHW involved (medium)	2.8

MHW, mental health worker; SIRP, Italian Society for Psychosocial Rehabilitation.

### Activity levels and evidence-based activities in the survey sample

Analyzing the data of the 2.255 cards related to structured activities, it emerged that 13.8% of them referred to evidence-based activities (355 cards). Evidence-based activities were recognized in literature with a clear evidence of efficacy (18, 19, 41); among these, we found within the facilities: cognitive remediation, psychoeducational interventions, social skills training and work related activities. Furthermore, we analyzed the distribution of EB activities among different facilities. Table 4 shows that the percentage of evidence-based activities was similar inside Italian facilities.

**TABLE 4:** Distribution of the evidence-based activities provided in the various types of facilities.

Facility	No.	EB	%
Semi residential facilities	829	133	16.0%
Mental health center	80	13	16.3%
Psychiatric diagnosis and care service facility	13	2	15.4%
Supportive housing	98	12	12.2%
Residential facilities	1,120	173	15.4%
Others	115	22	19.1%

## Discussion

The Survey outlines what has been implemented in Italy in terms of rehabilitation, albeit partial due to the spontaneous participation in the project: only 23.3% of the Italian Psychiatric Departments SIRP-related has attended. Since there’s nothing similar to this research in Italian psychiatric literature, this datum seems to be very significant. Considering the data collected from this partial presence of psychiatric services, it emerged that most of the facilities involved were residential (49.7%), whereas rehabilitation-focused facilities (semi-residential) were 36.8%.

In order to make the project reliable, shareable and repeatable, it was necessary to use efficient tools to conduct the analysis and to continuously review it (17, 55, 56), indeed 91.5% of facilities used at least PTI or PTR and 58.3% used both. To complete the frame, 68% of structures proved to use validated evaluation scales (57, 58), whereas 88.4% continuously reviewed the project; another significant information, looking at the aim of the survey. Regarding the activities performed during the investigation, they appeared to be conducted 62.3% in a group work (based on SISM categories and proved by SIRP ones); 18.5% individually and 19.2% in both modalities. Unfortunately evidence-based activities rate is under 13.8% of the total rehabilitation interventions, in line with the main European realities (44) and higher than US data, a clear proof that they’re still not widespread in the world, as well as in Italy (59). Through the analysis it emerged that 41.1% of group activities involved 6 to 10 patients per session and 58.9% 11 to 20. In psychiatric acute facilities patients per session were more than 20 and 2 to 5 were in Supportive Housing; the latter activities aimed more at supporting daily life, whereas in the other facilities the objective was to stimulate resocialization, interpersonal skills and physical activity. Sport is indeed one of the most proposed activities by MHC, followed by free time activities and psychoeducation for patients, that seems to be essential to support the patients at the onset of the disease and it also promotes mental health. Also cooking and gardening reached high percentages, respectively 55.6 and 55.2%, in all the facilities, due to the necessity to increase autonomy in patient’s everyday life. Following we can find art therapies, about expressive activities, which also have a strong tradition in Italy, representing 12.9% of the activity cards gathered at a national level. These data are significant because scientific evidence has demonstrated that group interventions foster socialization, improving the dynamics of imitation and modeling behaviors of the participating members (56).

Concerning individual activities, 6% of them aimed at stimulating interpersonal skills, 0.8% to social inclusion, whereof 4.7% of the projects were oriented to job placement and 3% were activities that also included family members. These small percentages are likely a consequence of the lack of staff resources, since these activities should be carried out individually (26, 60). These types of activities, that involve relatives, are strictly fundamental to provide an adequate quality to the cure, and therefore they should be compulsory, giving their importance (26, 61, 62).

As regards the location in which they took place, 60.4% of the activities were carried out inside the structures, whereas 20 to 35% outside. More than half of all the activities (61.5%) were developed within the structures continuously. These data are demonstrative of what in Italian rehabilitation keeps to lack: the percentage is still too low, since a great part of rehabilitation imply that most of the activities should be carried out in external contexts and in places that are part of the patient’s daily life (63, 64). As proof, the research resulted that only 0.5% of activities were carried out at the patient’s home, maybe for a lack of resources.

These data were extrapolated by the cards received, in which all rehabilitation activities were listed by the SIRP group: the majority came from residential and semi-residential facilities (62.5%).

### Methodological and operative critical issues

Considering the supplied material, the survey cards of the rehabilitation activities appeared understandable in its objectives, being complete and accessible. The abundance, completeness and pertinence of the answers obtained entailed also a comment on the clarity and relevance of the elaborated questions, which had been designed to leave a certain freedom of choice and expression to the compilers, that in this specific survey were the operators conducting the activities. This has allowed us to obtain an important amount of useful information to understand what is actually being done in the national rehabilitation reality, beyond the standardized definitions, established protocols and evidence of effectiveness.

Despite these strengths our study presents some limitations. The open-answer questions did not provide the categorization required for each activity, in order to allow a precise quantitative and qualitative definition of the described rehabilitation interventions. A critical point emerged in relation to the categorization of rehabilitation activities: for each of them it was necessary a free and open description, a codification by category, and a codification by SISM type. It was done to better define the detection of the activities that were carried out. Despite this, some inconsistent data appeared, which in some cases required telephone interviews with the operators who had completed the form and the revision of the database. The last matter to consider concerned the spontaneous and voluntary involvement in the project. Despite the participation was active, well received and interesting for the participating facilities, this manner of participation in the project obviously offered a lack of data, since this work is considered a survey and not a census.

## Conclusion

This survey on the rehabilitation activities in the Italian psychiatric services had the objective of outlining the state of the art of psychosocial rehabilitation in our country, taking into account that the participation of Italian facilities in this study was spontaneous and voluntary and therefore it can not represent a real and true census. Surely, it is useful to remember, that to date, there was no Italian data related to the subject of this thesis: the rehabilitation practices applied in the services are little known or shared, with the exception of some centers of excellence, which publish evidence from their own research protocols. With this survey we intended to present, at least partially, the “real-world” of rehabilitation in Italy so that we can lay the foundations for the definition of an updated, validated and shared network of what is implemented in the context of psychiatric rehabilitation. In fact, scientific research in the psychosocial field needs to be encouraged and implemented and requires the creation of a database involving the main active facilities. From the previously analyzed data, it emerges the need for greater dissemination even in non-university centers, of a rehabilitation based on evidence and proof of effectiveness, to integrate with other treatments in psychiatry, as suggested by several authors regarding personalized, effective and measurable treatment interventions (65). When we discuss psychiatric rehabilitation, it is complex to identify valid and unequivocal criteria to define the effectiveness of an intervention, because of the many intrinsic and extrinsic factors in the nature of mental disorders that can influence their outcome (65). The realization of this unique comprehension leads to a greater awareness of what is being done in Italy and becomes a starting point for the analysis of the critical points and strengths of Italian facilities in order to identify the correct path for the improvement of our services. In conclusion, it can be said that the data obtained are positive for some aspects of the rehabilitation interventions, in particular for the use of validated tools for the evaluation and revision of projects and for the trend to work on a team. The scarcity of evidence-based rehabilitation interventions applied in Italian psychiatric services is less encouraging, even though it is similar to international realities.

The study highlights how there are still important gaps in the sharing and employment of rehabilitation evidence-based practices in the national context and it underlines the need for new and further training among mental health workers in the field of psychiatric rehabilitation. In this regard, SIRP recently published a document on good practices and recommendations to keep in mind, with the ultimate goal of spreading the most current knowledge in this complex area (66).

## Data availability statement

The original contributions presented in this study are included in the article/Supplementary material, further inquiries can be directed to the corresponding author.

## Author contributions

CV, SB, and AV: conceptualization, methodology, and supervision. CA, DB, LF, AG, FR, and CV: data curation. CV and FR: investigation. CA, SB, DB, CV, and AV: writing, review and editing. All authors contributed to the article and approved the submitted version.

## References

[1] Andreou C Moritz S. Non-pharmacological interventions for schizophrenia: how much can be achieved and how? *Front Psychol.* (2006) 7:1289. 10.3389/fpsyg.2016.01289 27621717 PMC5002417

[2] Scull A. “Community Care”: historical perspective on deinstitutionalization. *Perspect Biol Med.* (2021) 64:70–81. 10.1353/pbm.2021.0006 33746131

[3] Feachem R. Health systems: more evidence, more debate. *Bull World Health Organ.* (2000) 78:715. PMC2560781 10916908

[4] Perera I. The relationship between hospital and community psychiatry: complements, not substitutes? *Psychiatr Serv.* (2020) 71:964–6. 10.1176/appi.ps.201900086 31896343

[5] Leucht S Kissling W Davis J. Second-generation antipsychotics for schizoP.hrenia: can we resolve the conflict? *Psychol Med.* (2009) 39:1591–602. 19335931 10.1017/S0033291709005455

[6] Jääskeläinen E Juola P Hirvonen N McGrath J Saha S Isohanni M A systematic review and meta-analysis of recovery in schizophrenia. *Schizophr Bull.* (2013) 39:1296–306. 10.1093/schbul/sbs130 23172003 PMC3796077

[7] Vita A Barlati S. Recovery from schizophrenia: is it possible? *Curr Opin Psychiatry.* (2018) 31:246–55. 10.1097/YCO.0000000000000407 29474266

[8] Correll C. Using patient-centered assessment in schizophrenia care: defining recovery and discussing concerns and preferences. *J Clin Psychiatry.* (2020) 81:MS19053BR2C. 10.4088/JCP.MS19053BR2C 32297720

[9] Weiden P. Redefining medication adherence in the treatment of schizophrenia: how current approaches to adherence lead to misinformation and threaten therapeutic relationships. *Psychiatr Clin North Am.* (2016) 39:199–216. 10.1016/j.psc.2016.01.004 27216900

[10] Sacchetti E Vita A. Poor adherence to antipsychotic medication in people with schizophrenia: diffusion, consequences and contributing factors. In: Sacchetti E Vita A Siracusano A Fleischhacker W editors. *Adherence to antipsychotics in schizophrenia.* Italia: Springer (2014). p. 1–84.

[11] Saito M. Improvement of medication adherence in psychiatry and mental health literacy education. *Yakugaku Zasshi.* (2021) 141:541–55. 10.1248/yakushi.20-00218 33790121

[12] Anthony W Cohen M Farkas M Gagne C. *Psychiatric Rehabilitation.* 2nd ed. Boston, MA: Center for Psychiatric Rehabilitation (2002). 10.1007/s10597-005-2649-6

[13] Corrigan P. *Principles and practice of psychiatric rehabilitation: an empirical approach.* 2nd ed. New York, NY: Guildford Press (2016).

[14] Galderisi S Rucci P Kirkpatrick B Mucci A Gibertoni D Rocca P Italian network for research on psychoses. interplay among psychopathologic variables, personal resources, context-related factors, and real-life functioning in individuals with schizophrenia: a network analysis. *JAMA Psychiatry.* (2018) 75:396–404. 10.1001/jamapsychiatry.2017.4607 29450447 PMC5875306

[15] De Berardis D De Filippis S Masi G Vicari S Zuddas AA. Neurodevelopment approach for a transitional model of early onset schizophrenia. *Brain Sci.* (2021) 11:275. 10.3390/brainsci11020275 33672396 PMC7926620

[16] Rössler W. Psychiatric rehabilitation today: an overview. *World Psychiatry.* (2006) 5:151–7. 17139342 PMC1636112

[17] Franck N. Psychiatric rehabilitation in schizophrenia. *Rev Prat.* (2021) 71:52–7. 34160939

[18] Mueser K Deavers F Penn D Cassisi J. Psychosocial treatments for schizophrenia. *Annu Rev Clin Psychol.* (2013) 9:465–97. 10.1146/annurev-clinpsy-050212-185620 23330939

[19] Vita A Barlati S. The implementation of evidence-based psychiatric rehabilitation: challenges and opportunities for mental health services. *Front Psychiatry.* (2019) 20:147. 10.3389/fpsyt.2019.00147 30949081 PMC6435578

[20] Fleischhacker W Arango C Arteel P Barnes T Carpenter W Duckworth K Schizophrenia: time to commit to policy change. *Schizophr Bull.* (2014) 40(Suppl. 3):S165–94. 10.1093/schbul/sbu006 24778411 PMC4002061

[21] Kem R Glynn S Horan W Marder S. Psychosocial treatments to promote functional recovery in schizophrenia. *Schizophr Bull.* (2009) 35:347–61. 10.1093/schbul/sbn177 19176470 PMC2659313

[22] Bond G Drake R. The critical ingredients of assertive community treatment: an update. *World Psychiatry.* (2015) 14:240–2. 10.1002/wps.20234 26043344 PMC4471983

[23] Marshall T Goldberg R Braude L Dougherty R Daniels A Ghose S Supported employment: assessing the evidence. *Psychiatr Serv.* (2014) 65:16–23. 10.1176/appi.ps.201300262 24247197

[24] Rog D Marshall T Dougherty R George P Daniels A Ghose S Permanent supportive housing: assessing the evidence. *Psychiatr Serv.* (2014) 65:287–94. 10.1176/appi.ps.201300261 24343350

[25] Bighelli I Rodolico A García-Mieres H Pitschel-Walz G Hansen W Schneider-Thoma J Psychosocial and psychological interventions for relapse prevention in schizophrenia: a systematic review and network meta-analysis. *Lancet Psychiatry.* (2021) 8:969–80. 10.1016/S2215-0366(21)00243-1 34653393

[26] Rodolico A Bighelli I Avanzato C Concerto C Cutrufelli P Mineo L Family interventions for relapse prevention in schizophrenia: a systematic review and network meta-analysis. *Lancet Psychiatry.* (2022) 9:211–21. 10.1016/S2215-0366(21)00437-5 35093198

[27] Turner D McGlanaghy E Cuikpers P van den Haag M Karyotaki E MacBeth A. A meta-analysis of social skills training and related interventions for psychosis. *Schizophr Bull.* (2017) 44:475–91. 10.1093/schbul/sbx146 29140460 PMC5890475

[28] Vita A Barlati S Ceraso A Nibbio G Ariu C Deste G Effectiveness, core elements, and moderators of response of cognitive remediation for schizophrenia: a systematic review and meta-analysis of randomized clinical trials. *JAMA Psychiatry.* (2021) 78:848–58. 10.1001/jamapsychiatry.2021.0620 33877289 PMC8058696

[29] Sackett D Rosenberg W Gray J Haynes R Richardson W. Evidence based medicine: what it is and what it isn’t. *BMJ.* (1996) 312:71. 10.1136/bmj.312.7023.71 8555924 PMC2349778

[30] Drake R Goldman H Leff H Lehman A Dixon L Mueser K lmplementing evidence-based practices in routine mental health service settings. *Psychiatr Serv.* (2001) 52:179–82. 10.1176/appi.ps.52.2.179 11157115

[31] Bassi M Ferrario N Ba G Delle Fave A Viganò C. Quality of experience during psychosocial rehabilitation: a real-time investigation with experience sampling method. *Psychiatr Rehabil J.* (2012) 35:447–53. 10.1037/h0094578 23276238

[32] Chiang M Reid-Varley W Fan X. Creative art therapy for mental illness. *Psychiatry Res.* (2019) 275:129–36. 10.1016/j.psychres.2019.03.025 30901671

[33] Chung J Woods-Giscombe C. Influence of dosage and type of music therapy in symptom management and rehabilitation for individuals with schizophrenia. *Issues Ment Health Nurs.* (2016) 37:631–41. 10.1080/01612840.2016.1181125 27192343

[34] Geretsegger M Mössler K Bieleninik Ł Chen X Heldal T Gold C. Music therapy for people with schizophrenia and schizophrenia-like disorders. *Cochrane Database Syst Rev.* (2017) 5:CD004025. 10.1002/14651858.CD004025 PMC6481900 28553702

[35] Guhne U Weinmann S Arnold K Ay E Becker T Riedel Heller S. Art therapies in severe mental illness: are they effective? *Nevernarzt.* (2012) 83:855–60. 10.1007/s00115-011-3472-7 22733379

[36] Koch S Riege R Tisborn K Biondo J Martin L Beelmann A. Effects of dance movement therapy and dance on health-related psychological outcomes. a meta-analysis update. *Front Psychol.* (2019) 10:1806. 10.3389/fpsyg.2019.01806 31481910 PMC6710484

[37] Ruiz M Aceituno D Rada G. Art therapy for schizophrenia? *Medwave.* (2017) 17(Suppl. 1):e6845. 10.5867/medwave.2017.6845 28112711

[38] Stuckey H Nobel J. The connection between art, healing, and public health: a review of current literature. *Am J Public Health.* (2010) 100:254–63. 10.2105/AJPH.2008.156497 20019311 PMC2804629

[39] Volpe U. *Arts therapies in psychiatric rehabilitation.* Berlin: Springer ed (2021).

[40] Volpe U Fiacchini D Magnotti R Diamare S Denti E Viganò C. “Le arti terapia nel contesto della riabilitazione psicosociale in Italia: una rassegna critica. *Psichiatr e psicoterapia.* (2016) 35:154–80.

[41] Vita A Corrivetti G Mannu J Semisa D Viganò C. Psychosocial rehabilitation in Italy today. *Int J Mental Health.* (2016) 45:15–23. 10.1080/00207411.2015

[42] Burti L. Italian psychiatry research 20 plus years later. *Acta Psychiatr Scand.* (2001) 410:41–6. 10.1034/j.1600-0447.2001.1040s2041.x 11863050

[43] Drake R Essock S. The science-to-service gap in real-world schizophrenia treatment: the 95% problem. *Schizophr Bull.* (2009) 35:677–8. 10.1093/schbul/sbp047 19502492 PMC2696380

[44] Rössler W Drake R. Psychiatric rehabilitation in Europe. *Epidemiol Psychiatr Sci.* (2017) 19:1–7. 10.1017/S2045796016000858 28100293 PMC6998639

[45] Kessler R Demler O Frank R Olfson M Pincus H Walters E Prevalence and treatment of mental disorders, 1990 to 2003. *N Engl J Med.* (2005) 352:2515–23. 10.1056/NEJMsa043266 15958807 PMC2847367

[46] Liberman R. Recovery from schizophrenia: form follows functioning. *World Psychiatry.* (2012) 11:161–2. 10.1002/j.2051-5545.2012.tb00118.x 23024668 PMC3449344

[47] Aloi M de Filippis R Grosso Lavalle F Chiappetta E Viganò C Segura-Garcia C Effectiveness of integrated psychological therapy on clinical, neuropsychological, emotional and functional outcome in schizophrenia: a RCT study. *J Mental Health.* (2018) 29:1–18. 10.1080/09638237.2018.1521948 30346226

[48] Deegan P. The importance of personal medicine: a qualitative study of resilience in people with psychiatric disabilities. *Scand J Public Health.* (2005) 33:1–7. 10.1080/14034950510033345 16214720

[49] Anthony W. Recovery from mental illness: the guiding vision of the mental health service system in the l 990s. *Psychosoc Rehabil J.* (1993) 16:11–23. 10.1037/h0095655

[50] Viganò C Borghetti S Casamenti R Borsani S Goffredi A Parabiaghi A Indagine sulle attività riabilitative in Lombardia. Un progetto della Società Italiana di Riabilitazione Psicosociale, sezione regionale SIRP-Lo. *Errepiesse.* (2012) 2:3–17.

[51] Ministero della Salute. Le Strutture Residenziali Psichiatriche. *Accordo Conferenza Unificata, 17 ottobre 2013.* Available online at: https://www.salute.gov.it/imgs/C_17_pubblicazioni_2460_allegato.pdf (accessed October 31, 2022).

[52] Ministero della Salute. Rapporto salute mentale. Analisi dei dati del Sistema Informativo per la Salute Mentale (SISM) anno 2016. (2016). Available online at: https://www.salute.gov.it/imgs/C_17_pubblicazioni_2731_allegato.pdf (accessed October 31, 2022).

[53] Teixeira C Santos E Abreu M Rogers E. Current status of psychiatric rehabilitation in Portugal: a national survey. *Psychiatr Rehabil J.* (2015) 38:263–7. 10.1037/prj0000144 25984735

[54] Roncone R Ussorio D Salza A Casacchia M. Psychiatric rehabilitation in italy: cinderella no more—the contribution of psychiatric rehabilitation technicians. *Int J Mental Health.* (2016) 45:24–31. 10.1080/00207411.2015.1119376

[55] Keepers G Fochtmann L Anzia J Benjamin S Lyness J Mojtabai R The American psychiatric association practice guideline for the treatment of patients with schizophrenia. *Am J Psychiatry.* (2020) 177:868–72. 10.1176/appi.ajp.2020.177901 32867516

[56] Vita A Dell’Osso B Mucci A (a cura di). Riabilitazione psichiatrica. *Manuale di clinica e riabilitazione psichiatrica. Dalle conoscenze teoriche alla pratica dei servizi di salute mentale.* (Vol. 2), Rome: Fioriti ed (2018).

[57] Allen N Couser G Bostwick J. Disability evaluation and treatment for patients with psychiatric disorders. *Mayo Clin Proc.* (2020) 95:1766–74. 10.1016/j.mayocp.2020.04.040 32753149

[58] Conti L. *Repertorio delle scale di valutazione in psichiatria.* Firenze: SEE Firenze ed (2000).

[59] Bond G Drake R. New directions for psychiatry rehabilitation in the USA. *Epidemiol Psychiatr Sci.* (2017) 26:223–7. 10.1017/S2045796016000834 27866508 PMC6998636

[60] Fadyl J Anstiss D Reed K Khoronzhevych M Levack W. Effectiveness of vocational interventions for gaining paid work for people living with mild to moderate mental health conditions: systematic review and meta-analysis. *BMJ Open.* (2020) 10:e039699. 10.1136/bmjopen-2020-039699 33122321 PMC7597525

[61] Song L. Correlates of community rehabilitation service utilization among persons with psychiatric disabilities. *Int J Soc Psychiatry.* (2021) 29:207640211036170. 10.1177/00207640211036170 34325552

[62] Datta S Daruvala R Kumar A. Psychological interventions for psychosis in adolescents. *Cochrane Database Syst Rev.* (2020) 7:CD009533. 10.1002/14651858.CD009533.pub2 32633858 PMC7388907

[63] O’Keeffe D Sheridan A Kelly A Doyle R Madigan K Lawlor E ‘Recovery’ in the real world: service user experiences of mental health service use and recommendations for change 20 years on from a first episode psychosis. *Adm Policy Ment Health.* (2018) 45:635–48. 10.1007/s10488-018-0851-4 29411173 PMC5999190

[64] Jaiswal A Carmichael K Gupta S Siemens T Crowley P Carlsson A Essential elements that contribute to the recovery of persons with severe mental illness: a systematic scoping study. *Front Psychiatry.* (2020) 11:586230. 10.3389/fpsyt.2020.586230 33329129 PMC7710894

[65] Starace F Baccari F Mungai F (A cura di). La salute mentale in Italia. *Analisi delle strutture e delle attività dei dipartimenti di salute Mentale.* Modena: Quaderni Italiani di Epidemiologia Psichiatrica (2017).

[66] Semisa D Bellomo A Nigro P Merlin S Mucci A (a cura di). *Raccomandazioni di buone pratiche in riabilitazione psicosociale per adulti.* Via Archimede: Giovanni Fioriti Editore (2022).

