# Supplementary material for: Psychiatric rehabilitation patterns in Italy: Results from the Italian Society of Psychosocial Rehabilitation (SIRP) survey
Source: Front Psychiatry. 2023 Feb 22;14:1130811. doi: 10.3389/fpsyt.2023.1130811 (PMC9992193; doi:10.3389/fpsyt.2023.1130811)
Supplement: Supplementary file 1 [file Data_Sheet_1.PDF]

SURVEY OF REHABILITATION ACTIVITIES IN THE DEPARTMENTS OF MENTAL HEALTH

Part I - Personal information

Region: **Province:**

Name of the service: identification code (ISTAT) **Operative Unit:** **Mental Health Department:**

Hospital / *ASL* :

User base (inhabitants):

Patients welcomed in the facility are mainly belonging to the user base (>80%): yes / no

Address:

Mobile:

e-Mail:

Part I General information regarding the service in which the forms are filled in:

(unique form for each service)

**Type of service**<br><br>(taken from National Information System 2001)	**Definition of an individualized rehabilitation project**<br>(in clinical folder)	**Evaluation with validated tools**	**Who carries out the evaluation**<br>(possible multiple choice)	**Mental Health Worker qualification**
o Mental Health Center<br>o Daily Center<br>o Day hospital<br>o Psychiatric Residential facilities<br>o With MHW 24h<br>o With MHW 12h<br>o With MHW 6h<br>o Supportive Housing<br>o Other residential service (indicate)<br>……………………………………<br>…………………………………….<br>o Hospital Psychiatric Service for Diagnosis and care (SPDC)<br>o<br>o Other (indicate)<br>…………………………………….<br>Bed N°:<br>users N° per day:	o **no**<br>o **yes,** in use:<br>o Individual Treatment Plan formalized by the sending team<br>o Therapeutic Rehabilitative Project formalized by the team of the facility<br>o both<br>o other…………………………<br><br>**Is there a periodic review of the project?**<br>□ no<br>□ yes<br>frequency:<br>1. standardized times<br>n. of months: ………………..<br>2. customized times	o NOT CARRIED OUT<br>o ONGOING<br>If carried out:<br>o **Psychopathological area**<br>Indicate which scales are used (e.g. BPRS, PANSS etc.)<br>………………………………………<br>………………………………………<br>………………………………………<br>………………………………………<br>o **Social functioning area**<br>Indicate which scales are used (e.g. GAF, DAS, LSP etc.)<br>………………………………………<br>………………………………………<br>…………………………………<br>o **Other**<br>Indicate which scales are used (e.g. QoL, VADO, Honos)<br>………………………………………<br>………………………………………<br>………………………………………<br>………………………………………<br>Ongoing ICF: yes - no	o psychiatrist<br>o psychologist<br>o nurse<br>o educator<br>o rehabilitation technician<br>o other……………<br><br>Is there a case manager?<br>o yes<br>o no<br>Does the case manager participate in the evaluation?<br>o yes<br>o no	Reference model of the MHW training carried out<br>o cognitive/behavioral<br>o psychoeducational<br>o systemic relational<br>o psychodynamic<br>o other……………………<br>Is there any supervision?<br>o yes<br>o no<br>If yes, what kind:<br>……………………………<br>frequency:<br>o regular<br>o sporadic<br>o as required

Part II of the questionnaire (please fill in a form for each activity carried out)
SINGLE REHABILITATION ACTIVITY DESCRIPTIVE SHEET
Part a.

NAME<br>and Brief DESCRIPTION of the REHABILITATION ACTIVITY	TYPE<br>OF INTERVENTION<br>(from SISM classification)	Conduction<br>MHW involved<br>(tick one or more boxes of interest and indicate the number	N° of users involved in the activity<br>(tick the box of interest)
--<br>--<br>--------------------------------<br>OPERATING METHODS (e.g. open or close group)<br>--<br>--<br>--<br>---------------------<br>contents:<br>--<br>--<br>--------------------------------<br>Techniques used:<br>--<br>--<br>--------------------------<br>Category n ° (use list A attached):<br>Estimated % distribution of participant diagnoses (class ICD10)<br>(es 295.x 50%; 296.x 30%; 301…% other x %, total 100%)	**Rehabilitation interventions addressed to the patient**<br>○ interventions with respect to basic, interpersonal skills<br>○ Individual<br>○ Group<br>○ Socialization interventions<br>○ Individual<br>○ group<br>○ Vacation Stay<br>○ Expressive therapies<br>○ Individual<br>○ group<br>○ Physical/Psychomotor activities<br>○ Practical-craft interventions<br>○ Vocational training interventions<br>**Social assistance intervention**<br>○ Daily life<br>○ Administrative and social problems<br>**Interventions for family members**<br>○ interview<br>○ psychoeducation<br>○ other<br>**Actions on the context:**<br>○ network interventions<br>○ interventions against stigma or other<br>○ other………..<br>○ **other typology**: ………………………………	○ nurse N°<br>○ professional health educator N°<br>○ rehabilitation technician N°<br>○ doctor N°<br>○ psychologist N°<br>○ art therapist N°<br>○ social assistant N°<br>○ volunteer N°<br>○ non-healthcare professional with specific expertise (e.g. artist, art master, coach, craftsman)<br>indicate: ……………………… n° ….<br>○ institutions/associations<br>indicate: …………………… n°…...<br>○ other<br>indicate: …………………… n°……..	○ 1<br>○ 2 - 5<br>○ 5 - 10<br>○ 10 - 20<br>○ ≥ 20

SINGLE REHABILITATION ACTIVITY DESCRIPTIVE SHEET
part b

1. PLACE.
The activity takes place on site: yes () no ();
if not, indicate place: () Domicile () Other place: ………………………………………………

2. FREQUENCY
The activity has a ……………. Frequency
• Regular: () bi-weekly () weekly () monthly () other ………

3. DURATION
The activity has a duration: continuous () to term () cyclical ()

At the time of the survey it is ongoing () it ended () it stopped ……………………………………………………………

4. Is the opinion of users systematically collected through a specific questionnaire? Yes () No ()

5. In the opinion of the operator, was the activity useful in order to achieve the objectives of the project? Yes () No ()

6. WERE DIFFICULTIES OBSERVED IN REALIZING IT? () YES () NO
IF YES, WHICH? (tick even more than one answer)

- ☐ Staff availability (operators)
- ☐ Personal availability (time)
- ☐ Availability of external collaborators
- ☐ Adequate space availability
- ☐ Availability of material (e.g. material for laboratories, economic resources)
- ☐ Stimulus and motivation of the patients involved
- ☐ Stimulus and motivation of the operators involved
- ☐ Other ____

Information about the person who filled in the form. Name, surname: *:……………………………………………………………………………………………*
Mobile:…………………………………………………….email…………………………………………………………………………….. date :

List of the completed activities

1 / name of the activity	
2	
3	
4	
5	
6	
7	
8	
9	
10	
11	
12	
13	
14	
15	
16	
17	
18	
19	
20	
